# Whole-Genome Resequencing Reveals Loci Associated With Thoracic Vertebrae Number in Sheep

**DOI:** 10.3389/fgene.2019.00674

**Published:** 2019-07-18

**Authors:** Cunyuan Li, Ming Li, Xiaoyue Li, Wei Ni, Yueren Xu, Rui Yao, Bin Wei, Mengdan Zhang, Huixiang Li, Yue Zhao, Li Liu, Yaseen Ullah, Yu Jiang, Shengwei Hu

**Affiliations:** ^1^College of Life Sciences, Shihezi University, Shihezi, China; ^2^College of Animal Science and Technology, Shihezi University, Shihezi, China; ^3^College of Animal Science and Technology, Northwest A&F University, Xianyang, China

**Keywords:** sheep genome, resequencing, selective-sweep analysis, variation in sheep vertebrae number, *VRTN* gene

## Abstract

The number of vertebrae, especially thoracic vertebrae, is an important economic trait that may influence carcass length and meat production in animals. However, the genetic basis of vertebrae number in sheep is still poorly understood. To detect the candidate genes, 400 increased number of thoracic vertebrae (T14L6) and 200 normal (T13L6) Kazakh sheep were collected. We generated and sequenced 60 pools of genomic DNA (each pool prepared by mixing genomic DNA from 10 sheep with the same thoracic traits), with an average depth of coverage of 25.65×. We identified a total of 42,075,402 SNPs and 11 putatively selected genomic regions, including the *VRTN* gene and the *HoxA* gene family that regulate vertebral development. The most prominent areas of selective elimination were located in a region of chromosome 7, including *VRTN*, which regulates spinal development and morphology. Further investigation indicated that the expression level of the *VRTN* gene during fetal development was significantly higher in sheep with more thoracic vertebrae than in those with a normal number of thoracic vertebrae. A genome-wide comparison between sheep with increased and normal numbers of thoracic vertebrae showed that the *VRTN* gene is the major selection locus for the number of thoracic vertebrae in sheep and has the potential to be utilized in sheep breeding in the future.

## Introduction

The mammalian spine is divided into different vertebral regions based on morphology and function along the head-to-tail axis, and these regions include the cervical (C), thoracic (T), lumbar (L), sacral (S), and caudal (Cd) regions (Schimandle and Boden, 1994). The number and morphology of vertebrae are usually conserved in a given species. However, variation in the number of thoracolumbar vertebrae regions has been observed in humans (Jones and German, 2014), pigs (King and Roberts, 1960), and mice (Zhu et al., 2012). Variation in the thoracolumbar region has been reported for commercial choice in pig breeding, and selection for such variation succeeded in raising the vertebral number and meat production of the bacon pig (Berge, 1948; Galis, 1999; Vasiliou et al., 2000; Hirose et al., 2013; Molofsky et al., 2013; Burgos et al., 2015; Yang et al., 2016; Brito et al., 2017; Duan et al., 2018). In addition, the number of vertebrae is genetically related to the number of teats, which contributes to improving the maternal capacity of livestock (Duijvesteijn et al., 2014; Lopes et al., 2014). There is rich variation in the number of vertebrae in sheep as well as pigs. Zhang et al. found that adding a thoracic or lumbar spine can increase the length of the Mongolian sheep spine by 2.4 or 3.5 cm, and multi-spine Mongolian sheep can affect the meat production of sheep by improving carcass length, carcass weight, length of the dorsal longus, and eye muscle area (Chen et al., 2012; Zhang and Si, 1997; Zhang et al., 1998). A study of European sheep breeds (Texel, Scottish Blackface, Texel × Mule, and Poll Dorset × Mule) by Donaldson et al. have shown that multi-vertebral traits can significantly increase the length of the body (and carcass) (Donaldson et al., 2013; Donaldson et al., 2014). The results of these studies are consistent with those of our previous reports showing that multi-vertebral variation may contribute to the performance of Kazakh sheep (Li et al., 2017), but one study showed that the number of rib pairs has little impact on meat production traits. However, an increased number of thoracic vertebrae will result in an increased number of rib pairs, which is a valuable economic trait in sheep due to the preference for lamb chops.

A large number of studies on variations in vertebrae number in pigs (Vasiliou et al., 2000; Fan et al., 2013; Hirose et al., 2013; Molofsky et al., 2013; Burgos et al., 2015; Yang et al., 2016; Yan et al., 2017; Duan et al., 2018) have shown that the *VRTN* gene strongly accelerates the development of additional thoracic vertebrae in pigs by enhancing the function of circadian oscillators. Further studies indicate that the 291-bp insertion in the upstream regulatory region of *VRTN* affects the expression level of this gene and leads to an increase in the number of porcine thoracic vertebrae (Fan et al., 2013). Rubin et al. used resequencing and selective-sweep analysis to find that the *NR6A1* gene controlled the number of lumbar vertebrae in pigs (Mikawa et al., 2011; Rubin et al., 2012). In addition, sequencing analysis of 10 breeds of pigs (three Western and seven Chinese breeds) showed that point mutations in *NR6A1* can affect the number of lumbar vertebrae in pigs (Yang et al., 2009). These studies have a positive impact on the selection of multi-spine large-scale pigs.

As an excellent local breed in China, Kazakh sheep has the characteristics of resistance to rough feeding, strong adaptability, and large body size. In order to meet the needs of production, herders consciously select larger individuals for breeding. In this breeding process, individuals with increased numbers of thoracic vertebrae may be subject to certain selection pressures due to their larger size. Information on the genetics of the vertebrae number in sheep is still lacking. To identify selective sweeps of candidate mutations related to an increased number of thoracic vertebrae of sheep, we performed genome-wide resequencing and a genome-wide comparison between increased and normal numbers of thoracic vertebrae in sheep, and then we functionally characterized the candidate genes. Our study not only screened the major genes controlling the number of thoracic vertebrae in sheep and promoted the development of new varieties of thoracic sheep but also enriched the genomic data of sheep.

## Materials and Methods

### Animals

We collected Kazakh sheep (8–10 months old) that were to be slaughtered in various regions of Xinjiang, China, and they were slaughtered at the slaughterhouse of Hualing Animal Husbandry Base in Urumqi. After the slaughterhouse used the electric shock to stun the sheep, these sheep were slaughtered using the neck bleeding method and then cut along the head-to-tail axis; all muscles were removed, leaving only the skeleton. The sheep were numbered, and the number of vertebrae was studied and recorded. The chest muscle tissues of 400 increased number of thoracic vertebrae (T14L6) and 200 normal (T13L6) Kazakh sheep were collected. For collecting spinal column samples (spinal tissue at the end of the thoracic vertebrae) during the fetal period, 12 pregnant ewes (uncertain thoracic number) were selected from a sheep population, and T13L6 or T14L6 fetuses were obtained from the slaughtered pregnant ewes. The fetuses were surgically removed in a sterile environment. The length of each fetus was measured and grouped based on size to approximate similar ages in T13L6 versus T14L6 samples (a total of 12 fetuses were selected; **Supplementary Table S1**). All of the samples were placed in cryovials and immediately frozen in liquid nitrogen. Then, they were transferred to a −80°C freezer until the genomic DNA or total RNA was extracted.

### DNA Library Construction and Sequencing

Genomic DNA was extracted from the frozen muscle tissues of Kazakh sheep using a kit following the manufacturer’s protocol. Forty pools (T14L6) and 20 pools (T13L6) of genomic DNA were prepared by mixing equally genomic DNA from 10 sheep with the same thoracic traits. Approximately 5 μg of DNA from each pool was used to construct a library and renamed (SG-1 to SG-40 composed the T14L6 group, and SG-41 to SG-60 were designated as the T13L6 group). These DNA samples were randomly sheared into 350-bp fragments using a Covaris crusher (Thermo Fisher, Waltham, USA). DNA library construction was performed using a TruSeq genomic library construction kit (Illumina, USA) as recommended by the manufacturer. Paired-end sequencing was performed using an Illumina HiSeq PE150 (Illumina, USA) after the constructed libraries were tested for quality using an Agilent 2100 (Agilent, California, USA). To ensure the quality of bioinformatics analysis, the raw data were filtered using a series of rigorous steps, which included the removal of joint sequences, sequences for which the number of undetected bases of single-end sequencing exceeded 10% of the total length of the sequence, and sequences with low-quality bases with a mass value of *Q* ≤ 5 in the single-end sequencing and that exceeded 50% of the total length of the sequence. The remaining high-quality clean reads were used in the subsequent bioinformatics analysis.

### Variation Detection and Annotation

To detect variations, we used BWA (http://bio-bwa.sourceforge.net/) (Jia et al., 2013) software to efficiently map the high-quality clean reads obtained as described above to the reference genome [GCF_000298735.2, National Center for Biotechnology Information (NCBI)] by recording their genomic positions. Next, we used SAMtools software to compare the results of the alignment after removing repeats, and a Bayesian model was employed to detect polymorphic sites (Li et al., 2009; Zhou et al., 2015). Furthermore, to obtain a high-quality mutation set, we filtered out single nucleotide polymorphisms (SNPs) with a sequencing error rate > 1% and intervals < 5 bp, and we retained those SNPs for which the reads had support values between one-third and two times the average depth. ANNOVAR software was used to functionally annotate the detected genetic variants (Wang et al., 2010) with gene-, region-, and filter-based annotations and for other functionalities.

### Selective-Sweep Analysis

To identify potential signals of selection that occurred during the breeding of T14L6 sheep, we calculated the *F*_ST_ value and *H*_P_ ratio of the variations observed in the whole genome and measured the differences between the two thoracic vertebral traits. The candidate selective sweeps were identified using the allele counts at the identified SNP positions by searching the genome for regions with a high fixation index (*F*_ST_) (Weir and Cockerham, 1984) and low pooled heterozygosity (*H*_P_) (Rubin et al., 2010; Stephan, 2010). We used the high-quality SNPs obtained as described above for further analysis to determine the signals of selection that led to changes in the number of thoracic vertebrae in sheep. First, allele counts were determined using custom-made scripts. Second, the *F*_ST_ values and *H*_P_ ratio were calculated in sliding 50-kb windows with a step size of 10 kb between sheep with 14 thoracic vertebrae and sheep with 13 thoracic vertebrae. In addition, the *F* *_ST_* was calculated using an estimator introduced by Karlsson et al., (2007). To avoid spurious selection signals, we discarded windows containing fewer than 10 informative sites from the subsequent analysis. The resulting *F*_ST_ values and *H*_P_ ratio were then *Z* transformed. Selective sweeps were extracted from the extreme tail of the distribution by applying cutoffs of *Z*(*F*_ST_) > 5 and *Z*(*H*_P_ ratio) > 5 (Axelsson et al., 2013). The regions detected by both methods were identified as the final selective sweeps (take the intersection of the windows of the *Z* score > 5 of the two methods as candidate regions). To avoid selective-sweep fragmentation, adjacent selective sweeps were combined. Both the *F*_ST_ and *H*_P_ ratio were co-screened for strong selection signals to screen for target genes.

### Analysis of the Expression Level of the *VRTN* Gene in Sheep Fetuses

The thoracic vertebral tissues were dissected from frozen sheep fetuses and used for total RNA extraction using TRIzol (Invitrogen, CA, USA) according to the manufacturer’s protocol. The amount and purity of RNA were detected using a Bioanalyzer 2100 system and an RNA 6000 Nano Kit (Agilent, CA, USA). cDNAs were synthesized from the purified RNAs using an RT-PCR kit (TaKaRa, Dalian, China). The sequence of the sheep *VRTN* gene (Gene ID: 101106594) was downloaded from the NCBI website, and Primer 5.0 software was used to design primers for this gene. The information on the primers is presented in **Supplementary Table S2**. For the expression level of the *VRTN* gene in sheep fetuses with different numbers of thoracic vertebrae, quantitative real-time polymerase chain reaction (RT-qPCR) was performed using SYBR Green (TaKaRa Biotech, Dalian) according to the manufacturer’s protocol. The expression level of the *VRTN* gene was normalized to that of linear β-actin (Peletto et al., 2011). Three independent replications were performed with triplicate samples. RT-qPCR was performed using the following reaction system: 10 μl of SYBR Premix Dimer Eraser (TaKaRa, Dalian, China), 2 μl of cDNA, 0.6 μl of upstream and downstream primers, and 6.8 μl of RNase-free ddH_2_O. RT-qPCR was performed using the following thermocycling conditions: an initial denaturation at 95°C for 30 s, followed by 55 cycles of 95°C for 10 s, 58°C for 10 s, and 72°C for 10 s, using a LightCycler 480 II system (Roche, Mannheim, Germany). The expression levels of these genes were determined using SYBR Green real-time PCR. Data were then analyzed using the equation 2ΔΔCt (Livek and Schmittgen, 2001).

## Results

### Whole-Genome High-Throughput Sequencing of Kazakh Sheep

**Figure 1:**
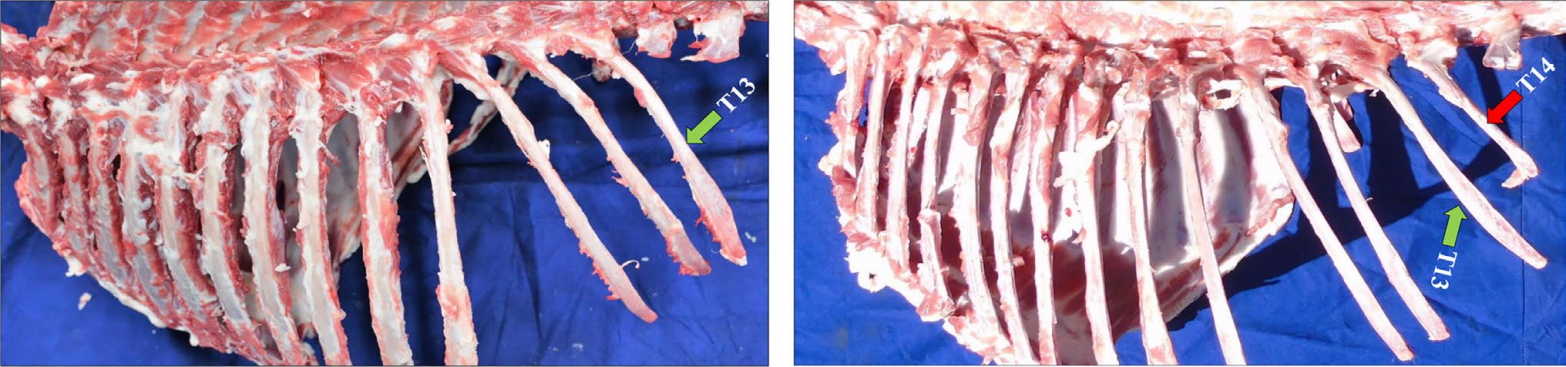
The morphology of the sample. There are two kinds of vertebrae in Kazakh sheep. T13L6 (left) and T14L6 (right). The green arrow indicates where the 13th thoracic vertebra is located, and the red one represents the position of the 14th thoracic vertebra.

To identify the candidate genes associated with increased numbers of thoracic vertebrae in sheep, 400 samples with the T14L6 trait and 200 samples with the T13L6 trait were collected throughout Xinjiang, China (**Figure 1**). Forty pools (T14L6) and 20 pools (T13L6) of genomic DNA were prepared by mixing genomic DNA from 10 sheep with the same thoracic traits. Sequencing of pools of genomic DNA was performed with 25.65-fold coverage of the sheep genome using an Illumina HiSeq PE150 system (**Supplementary Table S3**), which generated a total of 4,215.15 Gb of raw data. After inspecting the raw data for quality, we filtered out the low-quality and joint sequences, resulting in 4,207.35 Gb of clean data. The effective sequencing rate was 99.81%, indicating that the data had high quality and could be used for further in-depth analysis (**Supplementary Table S4**).

### Genome Variation Analysis

To detect variations in the T14L6 and T13L6 sheep genomes, we used BWA software to efficiently map the high-quality clean reads to the reference genome and obtained the genomic position of each read. Then, SAMtools software was employed to sort and remove duplicate sequences of these matching matches and identify polymorphic sites. After mapping the reads to the reference genome, we identified 42,075,402 SNPs (**Supplementary Table S5**), which is similar to the number of SNPs identified by current sheep resequencing studies (Pan et al., 2018; Bolormaa et al., 2019). A higher level of polymorphism was observed in the sheep genome of both T14L6 and T13L6 sheep. We filtered out the low-quality SNPs and used ANNOVAR software to functionally annotate the detected genetic variations to obtain a variation set.

### Selective-Sweep Analysis

**Figure 2:**
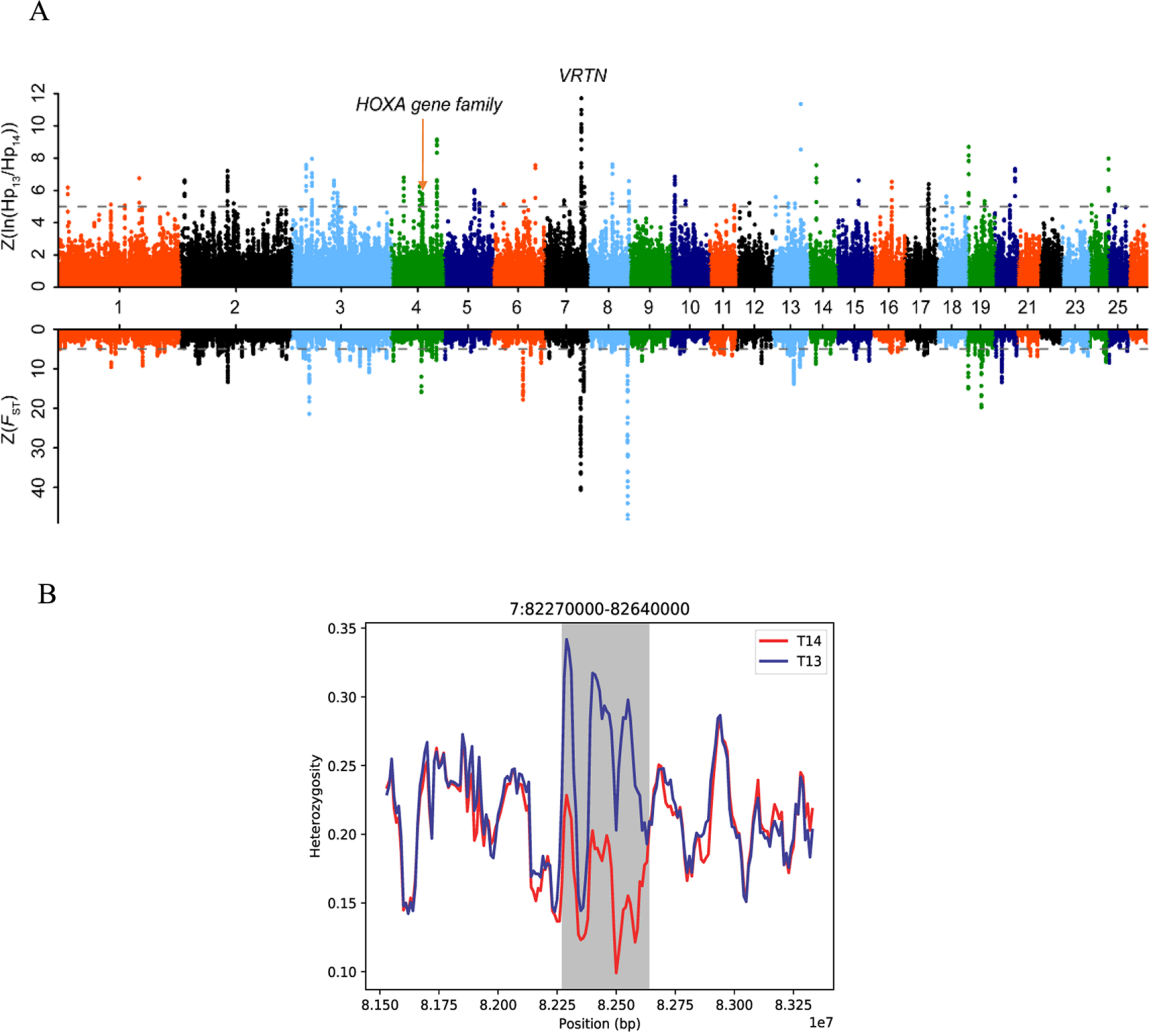
Selection analyses identified 11 candidate breeding regions. **(A)** *Z*(*F*_ST_) distributions plotted along sheep chromosomes 1–26 (chromosomes separated by color). The horizontal dotted line indicates the cutoff value used to extract outliers (*Z* > 5) (top part). Draw the *Z* (ln(*H*_P_13/*H*_P_14)) distribution along goat autosomes 1–26 (chromosomes separated by color). The horizontal dotted line indicates the cutoff value (*Z* > 5) used to extract outliers (bottom part). **(B)** Pooled heterozygosity (*H*_P_) in 45-kb sliding windows with 10-kb steps in T14 (red line) and T13 sheep (blue line) from 82.27 to 82.64 Mb on chromosome 7.

To detect recently strongly selected signals, we searched the sheep genome for areas with reduced heterozygosity (*H*_P_) and increased genetic distance (*F*_ST_). To separate the selected true signal from the signal of the randomly fixed large genomic region during sheep breeding, we set the threshold for detecting the true signal to be greater than 200 kb. A total of 11 [*Z*(*F*_ST_) > 5 and *Z*(*H*_P_ ratio) > 5] putative selected genomic regions distributed across eight chromosomes containing 28 genes were identified (**Table 1**, **Figure 2**, **Supplementary Figure S1**, and **Supplementary Table S6**). Interestingly, we found that these genes included *VRTN* and the *HoxA* gene family, which regulate vertebral development (Kostic and Capecchi, 1994; Horan et al., 1995; Yang et al., 2016). We further focused on the region with a strong selection signal (with the highest *H*_P_ ratio and second *F*_ST_ values) on chromosome 7 [82,260,000–82,660,000 bp, *Z*(*H*_P_ ratio) = 11.82 and *Z*(*F*_ST_) = 41.44] (**Figure 2A**). This region harbors the *VRTN* gene, which is related to spinal development and morphology (**Supplementary Table S7**). The results indicated that the heterozygosity of the *VRTN* gene in the T14L6 sheep was significantly lower than that in the T13L6 sheep (**Figure 2B**, **Supplementary Table S8**, and **Supplementary Table S9**).

**Table 1:** Genomic regions identified as candidate selective sweeps. The maximum statistics for *Z*-transformed *F*_ST_ and *Z*-transformed *H*_P_ ratio (*H*_P_13/*H*_P_14) in each region.

Chr	Start	End	Max(*Z*(*F*_ST_))	Max(*Z*(*H*_P_ ratio))	Candidate genes
2	103950000	104070000	6.960778	7.281002	*MTMR9*, *LOC105608545*, *LOC101118164*
3	31880000	31980000	7.720198	7.653394	NCOA1
3	102450000	102570000	6.669204	5.888367	INPP4A
4	68790000	68890000	6.235270	5.845450	*HoxA13*, *LOC105606592*, *HoxA11*, *HoxA10*, *HoxA9*, *LOC105606599*, *HoxA3*, *HoxA5*, *HoxA4*
4	101840000	101930000	8.030730	9.242812	TRIM24
7	82270000	82640000	41.194798	11.822053	*BBOF1*, *ALDH6A1*, *LIN52*, *VSX2*, *ABCD4*, *VRTN*, *SYNDIG1L*, *NPC2*
7	89280000	89400000	16.083480	6.280792	TSHR
8	52220000	52280000	5.782953	5.851114	CEP162
14	15240000	15320000	9.035530	7.638793	ITFG1
17	51180000	51250000	6.299107	5.510096	LOC106991703
19	2130000	2240000	15.327960	8.775768	LOC105603333

### Analysis of Expression Levels of the *VRTN* Gene

**Figure 3:**
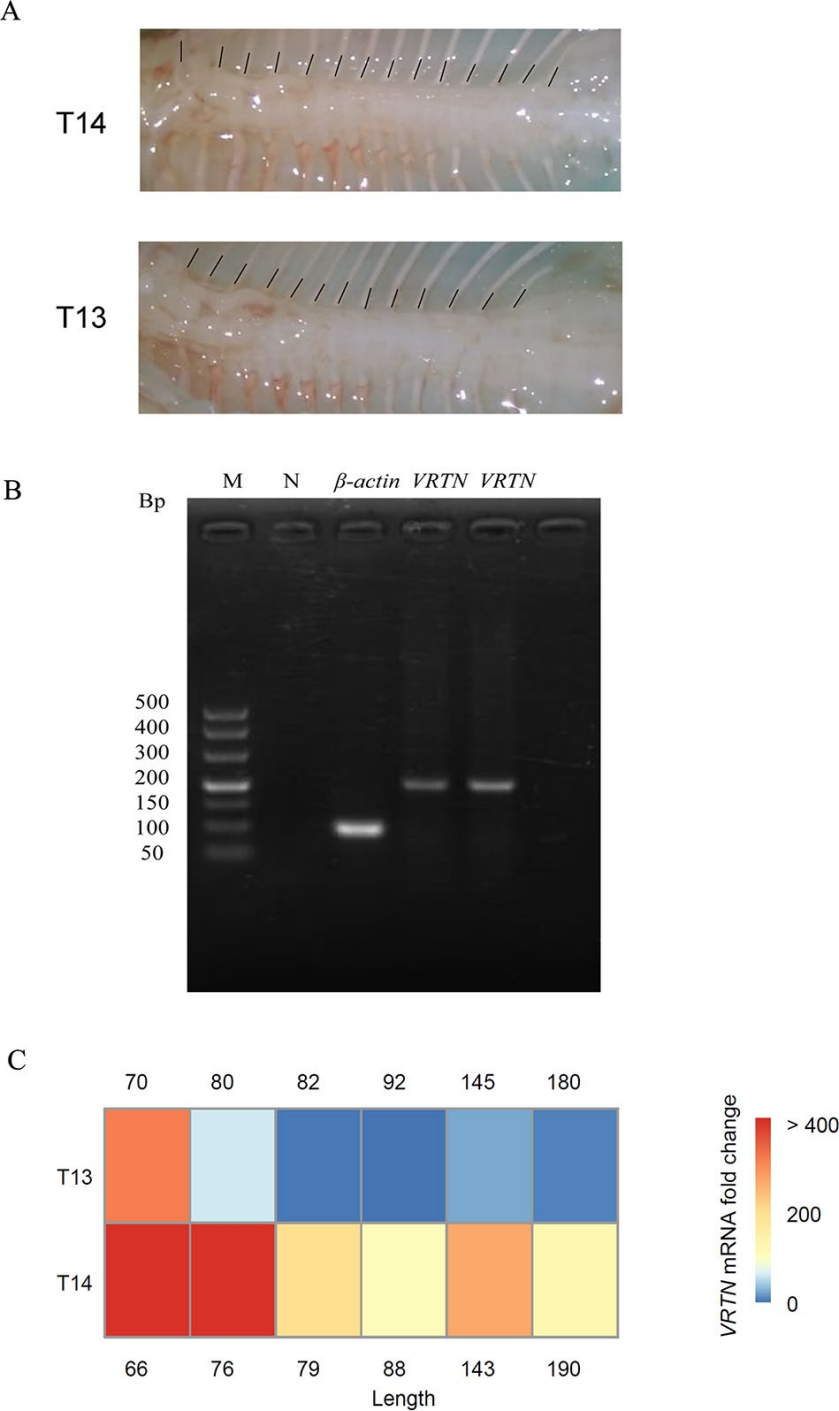
Analysis of *VRTN* gene expression levels in different fetal stages for two spine traits. **(A)** Legend of spine traits during fetal development: T14 (top) and T13 (bottom). **(B)** Reverse transcription Polymerase Chain Reaction (RT-PCR) amplification of β-actin and *VRTN* (on the left is T13 and on the right is T14, both from the same period of fetal sheep). The order of electrophoresis is marker (TaKaRa DL500; 500, 400, 300, 200, 150, 100, and 50 bp), negative control, β-actin, and *VRTN*. **(C)** Heat maps of *VRTN* gene expression in T13 (top) and T14 (bottom) fetuses (*p* < 0.05). Red indicates high expression, and green indicates low expression. β-Actin was used as a control. The numbers on either side represent the length of the fetus. The unit is mm.

We analyzed the expression level of *VRTN* in thoracic vertebral tissues at different times during sheep fetal development by RT-qPCR. Interestingly, we found that the expression level of the *VRTN* gene in T14L6 thoracic vertebrae (**Figure 3A**) was significantly higher than that in T13L6 thoracic vertebrae (*p* < 0.05) (**Figures 3B, C**). In addition, *VRTN* expression levels were inversely correlated with the fetal development stage of sheep. Our results are consistent with the results of previous studies reporting that *VRTN* expression levels are positively correlated with the number of thoracic vertebrae in mice and pigs but negatively correlated with fetal developmental stages (Duan et al., 2018). These results also help to show that VRTN is the major gene regulating the number of thoracic vertebrae in sheep.

## Discussion

In animal husbandry production, animals exhibiting good traits have been historically selected for further breeding. Therefore, artificial breeding of animals with superior traits for an extended period of time results in a decrease in the number of polymorphisms within that particular species (Gibbs et al., 2009), and the identification of these selected regions may facilitate mapping trait-related genomic regions. To identify the selected signals associated with an increase in the number of thoracic vertebrae in sheep, large-scale resequencing of Kazak sheep with T14L6 and T13L6 traits was performed in this study. Selective-sweep analysis showed that 11 putative selected regions in the sheep genome were associated with the increase in the number of thoracic vertebrae. These regions contain genes that are related to the development and growth of thoracic vertebrae. Among these genes, seven genes belonging to the *Hox* family have higher selection signals, and mutations in this gene family can lead to morphological imbalances and spinal variations in animals (Mallo et al., 2010). There is strong evidence that the *TSHR* gene can regulate bone morphogenesis and bone density (Van et al., 2008), and *TSHR* knockout mice display high-turnover osteoporosis (Jiang et al., 2006). The *ALDH6A1* gene can regulate the development of the spinal cord, and the loss of this gene causes stunting (Vasiliou et al., 2000; Molofsky et al., 2013). The *VRTN* gene regulates the formation and development of thoracic vertebrae (Vasiliou et al., 2000; Hirose et al., 2013; Molofsky et al., 2013; Burgos et al., 2015). The *NPC2* and *INPP4A* genes play important roles in regulating the normal functioning of spinal cord neurons (Sasaki et al., 2010; Walkley and Suzuki, 2004). These results also show that our sequencing has accurately screened genomic regions that are associated with variations in the number of thoracic vertebrae in sheep.

By studying the region of chromosome 7 that exhibited the highest selection signals, we have associated the *VRTN* gene with the morphology and number of thoracic vertebrae in sheep. Analysis of genome-wide heterozygosity has indicated that the base heterozygosity of the *VRTN* gene in T14L6 sheep is significantly lower than that in T13L6 sheep. Fan et al., (2013) and Zhang et al., (2015) have shown that the *VRTN* gene, as a novel transcription factor, can affect the number of thoracic vertebrae in pigs. In this study, we used RT-qPCR to quantitatively study the *VRTN* gene in fetal Kazak sheep thoracic vertebrae. The expression level of the *VRTN* gene in sheep fetuses with more thoracic vertebrae was significantly higher than that in sheep fetuses with normal vertebrae numbers (*p* < 0.05). Specific mechanisms and causal mutational genotypes require more in-depth and meticulous research in the future.

In summary, we conducted a genome-wide comparison between increased and normal numbers of thoracic vertebrae to identify genes that affect spinal development and morphology. Consistent with a previous study in pigs, the *VRTN* gene was related to the number of thoracic vertebrae in sheep. Our results have indicated that the *VRTN* gene may be a new candidate gene for breeding sheep with more thoracic vertebrae.

## Data Availability

All the sequences have been deposited in the Sequence Read Archive (https://www.ncbi.nlm.nih.gov/sra) with the accession codes PRJNA479525. In addition, all the data are also available from the corresponding author on reasonable request.

## Ethics Statement

All experimental operations and surgical procedures for the sheep involved in this study were performed in accordance with the “Regulations for the Management of Laboratory Animals” (Chinese State Council No. 676; revised in March 2017) and were carried out with approval from the Animal Protection and Use Committee of Shihezi University (SU-ACUC- 08032).

## Author Contributions

CL, XL, ML, YJ, WN, and SH conceived and designed the experiment and analyzed and interpreted the data. CL, XL, ML, YJ, WN, and SH wrote the manuscript. CL, XL, YX, MZ, HL, BW, YZ, LL, RY, and YU collected samples and data. CL, XL, and ML participated in RNA extraction and analysis. All authors read and approved the final manuscript.

## Funding

This work was supported by the National Natural Science Foundation of China (NSFC) (31660644, 31660718, 31822052 and 31572381), Young innovative talents (CXRC201603 and 2016BC001), the Recruitment Program of Global Young Experts (1000Plan).

## Conflict of Interest Statement

The authors declare that the research was conducted in the absence of any commercial or financial relationships that could be construed as a potential conflict of interest.
