# Supplementary material for: Whole-Genome Resequencing Reveals Loci Associated With Thoracic Vertebrae Number in Sheep
Source: Front Genet. 2019 Jul 18;10:674. doi: 10.3389/fgene.2019.00674 (PMC6657399; doi:10.3389/fgene.2019.00674)
Supplement: Supplementary file 2 [file DataSheet_1.pdf]

Fig. S1

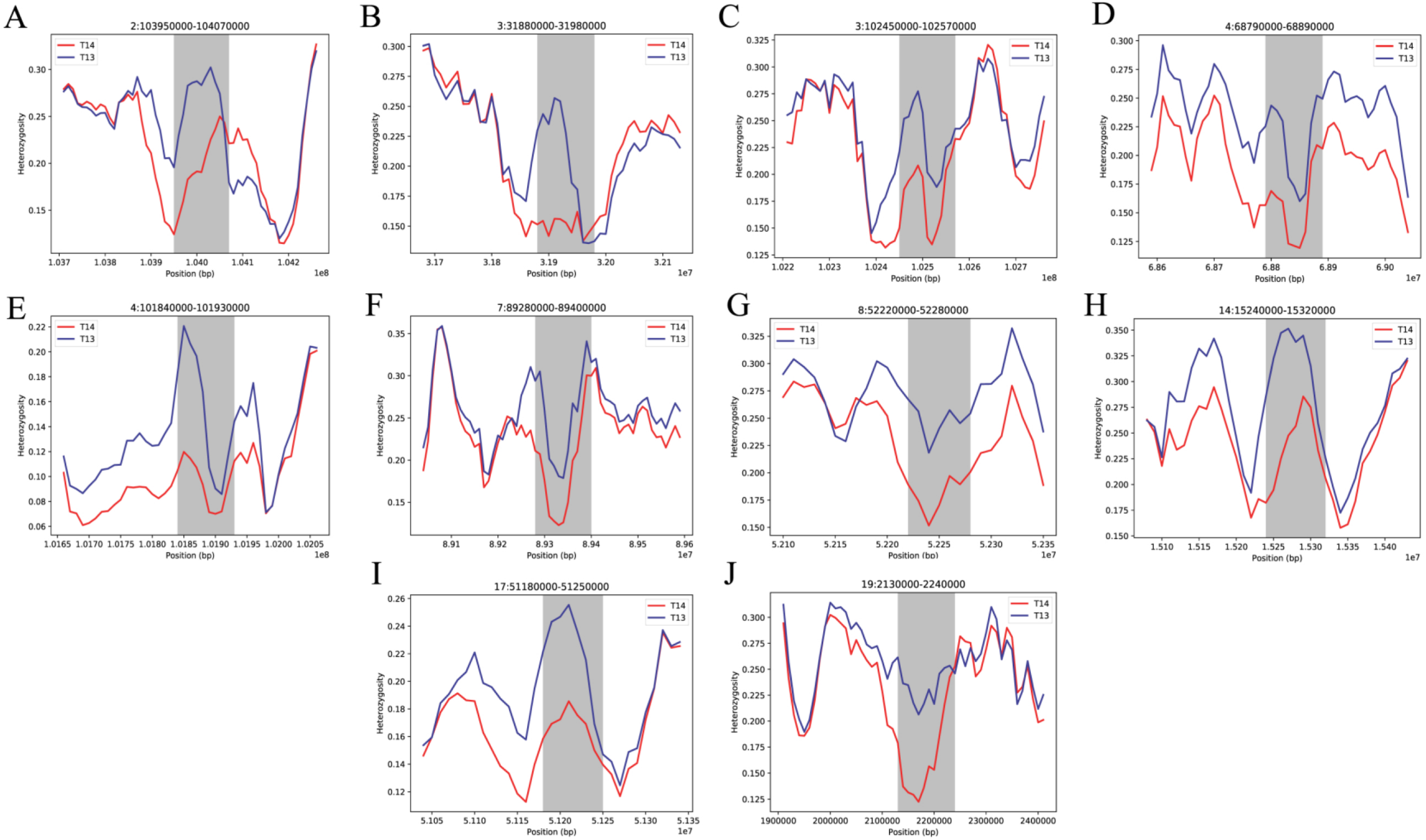

Figure S1. The pooled heterozygosity (HP) of the selected regions in a 45kb sliding window with a 10 kb step. (A) From 103.95 to 104.07 Mb on chromosome 2. (B) From 31.88 to 31.98 Mb on chromosome 3. (C) From 102.45 to 102.57 Mb on chromosome 3. (D) From 68.79 to 68.89 Mb on chromosome 4. (E) From 101.84 to 101.93 Mb on chromosome 4. (F) From 89.28 to 89.40 Mb on chromosome 7. (G) From 52.22 to 52.28 Mb on chromosome 8. (H) From 15.24 to 15.32 Mb on chromosome 14. (I) From 51.18 to 51.25 Mb on chromosome 17. (J) From 21.30 to 22.40 Mb on chromosome 17. T14 (red line) and T13 sheep (blue line).
